# Opinion: Advancing science in support of sustainable bio-innovation: 16th ISBR Symposium – in memory of Professor Alan Raybould

**DOI:** 10.3389/fbioe.2025.1593891

**Published:** 2025-04-25

**Authors:** Alan Gray, Monica Garcia-Alonso, Karen Hokanson, Andrew Roberts, Jörg Romeis, Joe Smith

**Affiliations:** ^1^ UK Centre for Ecology and Hydrology (UKCEH), Wallingford, Oxfordshire, United Kingdom; ^2^ Estel Consult Ltd., Berkshire, United Kingdom; ^3^ Agriculture & Food Systems Institute, Washington, DC, United States; ^4^ Research Division Agroecology and Environment, Agroscope, Zürich, Switzerland; ^5^ Independent Researcher, Canberra, ACT, Australia

**Keywords:** Alan Raybould, problem formulation, GMO risk assessment, ISBR symposium, biosafety

## Introduction

Just months before the 16th Symposium of the International Society for Biosafety Research (ISBR) took place in St. Louis, MO (May 2023), the ISBR community suffered a shattering loss with the death of our dear friend and colleague Alan Raybould. Among Alan’s innumerable contributions to the field of risk assessment and biosafety, he was an active contributor to ISBR over the course of his career, serving on the Board of Directors and as a member of the program committee, member and chair of the publication committee, contributing to symposium planning, chairing sessions and, as he was always known to do, delivering many notable and thought-provoking presentations at the symposia. The loss of Alan has left a hole in our community that simply cannot be filled. During the 16th ISBR Symposium, Professor Alan Gray, former President of ISBR, who was an advisor, colleague, and longtime close friend of Alan, shared a tribute. We honour the memory of Alan Raybould by sharing that tribute as part of this Research Topic produced after the Symposium.

## The life and work of Professor Alan Raybould (1962–2022) - A tribute

As presented by Professor Alan Gray at the 16th Symposium of the International Society for Biosafety Research, St. Louis, Missouri, 3 May 2023.

“First I would like to thank the Board of the ISBR for inviting me to pay this tribute to Alan – I am deeply honoured to do so. I will begin by recounting my personal experiences of Alan’s life and work and will then review his contribution as a scientist and scholar. Finally, I will attempt to capture something of Alan’s character and personality.

### Alan Raybould–Colleague and friend

I first met Alan in 1985 when my friend Mike Lawrence of the University of Birmingham’s Department of Genetics suggested that we share a PhD student to tackle a project on the topic of population genetics. He came to us with, to quote his professor, “the best first in Manchester in a decade”. He worked on the evolutionary origins of the grass *Spartina anglica,* doing the lab work in Birmingham (in the dear old days of isoenzyme electrophoresis) and the field work from Furzebrook (then a research station of The Institute of Terrestrial Ecology). I had moved to Furzebrook, which is in Dorset in southern England, a few years before and had begun to collect some type material of this famous allopolyploid invasive species, the site of its origin being nearby in Southampton harbour; but other commitments had prevented me from working on it. A copy of Alan’s simply brilliant thesis exploring genetic variation in the putative parental species, the F1 hybrid and the allopolyploid has pride of place on my bookshelves.

After a brief post-Doc in Birmingham Alan came to work in my group at ITE Furzebrook in 1990 and thus began more than 3 decades of exciting collaboration and wonderful friendship. We were researching genetic variation in natural populations of plants in relation to factors such as population size and isolation, breeding systems, life history traits and gene flow–mainly from a conservation genetics perspective. So when the GM crops debate took off we were well positioned to make a contribution to understanding the potential impacts of gene flow between GM crops and their wild relatives. In fact, one of our early papers in which we combined Alan’s knowledge of genetic modification and my knowledge of the ecology of wild plants to look at the implications of hybridisation between modified crops and their United Kingdom relatives (Raybould and Gray, 1993) remains his second most cited publication.

These were exciting times for our group. We expanded our work on gene flow and population genetics to look at the wild relatives of some United Kingdom crops, most notably species of *Brassica* and *Beta* (and also some grasses). Among other areas, we studied hybridisation rates and effects and the prospects of ecological release by the transfer of ecologically relevant, fitness, traits to wild populations. Our models for this included virus resistance in wild *Brassica oleracea* and *Brassica rapa* and the role of the bolting gene in *Beta maritima*. To understand the role of virus resistance in wild Brassica populations required collaboration with the virologists in our own Institute’s Oxford lab and with other specialist plant breeding institutes as well as University groups, as indeed did much of our research at that time.

And this was when we became aware of Alan’s great gifts as a collaborator. His curiosity and fascination for science, the breadth of his intellect and his ability to forge friendships made him a natural collaborator (in my experience not necessarily a common trait in the intellectually gifted). He collaborated and published with a huge range of people in the broad field of Environmental Risk Assessment, in research institutes, crop breeding organisations and universities in the United Kingdom and abroad. Many of you are in this room. Such was his gift for understanding and making a contribution to a wide range of problems that, during this period, people in our lab and institute (which became the United Kingdom Centre for Ecology and Hydrology) working in other areas sought him out with their problems.

Alan’s research with our molecular ecology group included a wide range of topics and collaborations. Working with the conservation genetics group he published “Conserving genetic variation in *Lobelia urens* populations” (a rare heathland plant) and “Status of *Rumex rupestris* populations, survival and genetic diversity” (a threatened shore dock). His work on gene flow included theoretical and empirical ways of defining and measuring gene flow in *B. maritima* (inferring patterns of dispersal from allele frequency data using molecular markers to estimate gene flow with distance and regional-scale estimates of transgene spread from oilseed rape (also using remote sensing).

His published work on viruses in *Brassica* species includes studies of spatial distribution of viruses in natural populations of *B. oleracea,* the effect of turnip mosaic virus and turnip yellow mosaic virus on survival growth and reproduction in *B. oleracea*, viruses in *B. rapa* and heritable variation for control of turnip mosaic virus and cauliflower mosaic virus in *B. oleracea*. Further work on fitness and selection in wild *B. oleracea* produced a series of papers on plant-herbivore interactions including the ecological genetics of aliphatic glucosinolates and host plant location and herbivory in the cabbage seed weevil *Ceuthorynchus assimilis.*

### Alan Raybould–Scientist and scholar

I imagine that a lot of Alan’s earlier work will be new to an ISBR audience. But its range and brilliance will, I’m sure, not surprise you. It foreshadows the pattern of his later work and contribution to risk analysis, risk assessment and the role of science in decision making. That work also involved collaboration with a wide range of actors, many of whom are in this room. First, however, I would like to trace an aspect of Alan’s thinking and development whilst he was still with us.

By 1995 he had become Head of the Molecular Ecology group and, since my own responsibilities had increased, our interactions were less frequent and mainly consisted of chats in the lab, at meetings, coffee and in the pub. It was clear that he was becoming increasingly interested in the scientific method, in philosophy of science and in hypothesis-based approaches. He was reading the work of and fascinated by Karl Popper (as some will know, Alan and his wife Clare’s beloved dog is called Popper). We began to see before he left us the thinking that prevailed upon us not to continue to accumulate data without first defining and clarifying the perceived risks. We began to hear in his talks about ‘black swans’ and ‘buckets and searchlights’ (Raybould, 2010a) and the various metaphors he employed to urge us all to stop accumulating the ecological data it is ‘nice to know’ but to focus on that which will help us answer the ‘need to know’.

The titles of two of Alan’s single author papers (in 2004 and 2010) reflect this viewpoint.

“A decade of gene flow research: improved risk assessment or missed opportunities?” (Raybould, 2004)

“Reducing uncertainty in regulatory decision -making for transgenic crops: more ecological research or clearer environmental risk assessment?”(Raybould, 2010b)

Papers with two rhetorical questions which his life’s work went on to answer so effectively.

In a perverse way all the fascinating discoveries about ecology and selection in natural populations he made at the Centre for Ecology and Hydrology may have paved the way for the focus of his later highly influential contribution. I must conclude that my major influence on the thinking and development of this brilliant man was to show him how not to do an environmental risk assessment! Anyway by 1999 it was clear that his fascination with the issues of risk assessment and his love of applied and socially relevant problems would lead him to other things and specifically agricultural biotechnology, and in 2001 he joined Syngenta.

In 2019 Alan was appointed as the Chair of Innovation in the Life Sciences in the University of Edinburgh and a new phase of his life began in which he added “teacher” to his list of gifted skills. By all accounts he was exceptional at that too. In 2020 he was appointed to the Advisory Committee on Releases to the Environment (ACRE), the UK’s independent body which provides statutory advice to the United Kingdom government on the risks to human health and the environment from the release of genetically modified organisms (GMOs). This is the same body to which I was appointed in 1994 and Chaired from 1999 to my retirement in 2003. I have recently learned that he was due to be made Vice Chair. I cannot tell you how delighted and proud I was on hearing that news.

Alan and I kept in touch, not only as friends but also meeting and working together in conferences, seminars and workshops around the world. In fact in 2007, after I had retired, Alan persuaded me to join him and others at a workshop in Washington on problem formulation (Raybould, 2006) which rekindled my interest in risk assessment and converted me to adopt his approach–the former student teaching the former teacher! I was privileged to be involved in several of the many workshops to which he made a key contribution and in which I learned so much from him.

There is absolutely no doubt that Alan’s life and work were transformational. His contribution to biosafety research, environmental risk assessment, the application of problem formulation and policy-based decision-making, among other areas, has been immense, profound and game-changing. He was a prolific writer–of his more than 180 papers, articles and reports, more than 100 were concerned with this area. As when he was at the Centre for Ecology and Hydrology these publications demonstrate his skills as a collaborator (working with many of the ISBR family) but also his originality, clarity of thought, rigour and common-sense pragmatic approach to the issues he tackled. His work gained international recognition and influenced regulatory approaches around the world.

I hesitate, because I am probably not the most qualified person, to evaluate the impact of this later body of work. I quote below some of the things said about his influence in the various tributes paid to him.

“a giant in the world of biodiversity research and risk assessment of new technologies in agriculture….a leading force in improving the way new technologies are evaluated and regulated around the world”

“a wonderful scientist who managed to bring clarity to any topic he approached. He fundamentally changed the way the risk assessment for genetically modified organisms was viewed by leading the discussion away from endless data generation to a targeted hypothesis driven approach informed by a policy context”

“he left a legacy of scientific work that will not only inspire new generations of regulatory folk but also is adaptable enough to cover new technologies”

“well recognised as a true leader and mentor in his field. His contributions have already had great impact and will continue to do so, as his published works will guide science and regulation for many years”

These are just a few of the many tributes to his work that say more powerfully than I could what a huge impact he has had. Hopefully these and countless other memories we have of a wonderful and gentle man, together with his magnificent legacy of words and ideas, will help to sustain us as we face a future without him.”
